# Infected hip prosthesis in patient with suspected Covid-19 infection

**DOI:** 10.1186/s42836-020-00058-0

**Published:** 2021-02-02

**Authors:** A. Cosentino, G. Odorizzi, W. Berger

**Affiliations:** F. Tappeiner Hospital, via Rossini 5, 39012, Meran, BZ Italy

**Keywords:** Prosthetic joint infection, Hip infection, COVID-19 pandemic

## Abstract

**Background:**

Infections following arthroplasty are one of the major risks during this type of surgery. Moreover, the outbreak of coronavirus disease 2019 (COVID-19), caused by SARS-CoV-2 (Severe Acute Respiratory Syndrome CoronaVirus Disease 2), has developed into an unprecedented pandemic, posing enormous pressure on health-care providers around the world.

**Case presentation:**

Four and half years after right hip arthroplasty, the patient came back to our attention with pain at the same hip. The instrumental examinations showed signs of cup detachment. After carefully analyzing the case, we decided to perform a sterile aspiration of the hip in the operating room under C-arm fluoroscopy. Microbiological examinations showed positivity for *E. coli*. The patient underwent surgery by which the prosthesis was removed and a spacer was implanted. A therapy with Cefotaxim 2 g three times a day for 6 weeks was then set, and then a total arthroplasty was performed. During this period, the COVID-19 pandemic occurred and therefore the patient received nasal-throat swabbing two times, and both yielded negative results. However, 1 week after the final surgery, his respiratory conditions deteriorated and chest X-ray and CT scan showed images of ground-glass opacification patterns (GGO). Due to the clinical symptoms and the characteristic images of the instrumental examinations, the patient was transferred to an observation ward. Thereafter, two more swab tests gave negative results. The patient was then transferred to the ward for patients with typical symptoms of COVID-19 but with negative swab tests for 2 weeks and was subsequently discharged home.

**Conclusion:**

The purpose of this case report was to point out the correct treatment of a PJI after the outbreak of COVID-19. Despite the ongoing COVID-19 pandemic, the guidelines in the case of periprosthetic hip infection further confirmed the correct management of the patient.

## Background

Knee and hip replacements are two most commonly performed elective operations. For most of the patients, joint replacement relieves pain and helps them to live more fulfilling and active lives. Nonetheless, no surgical procedure is without risks. A small percentage of patients undergoing hip or knee replacement (roughly about 1 in 100) may develop infection after the operation. Joint replacement infections may occur in the wound or deep around the artificial implants.

A new coronavirus disease, known as Severe Acute Respiratory Syndrome Coronavirus 2 (SARS-CoV-2) causing coronavirus disease 2019 (COVID-19), hit in December 2019 [1]. On March 11th 2020, the World Health Organization (WHO) declared the disease a pandemic. By then, more than 118,000 individuals were infected in 113 countries and regions. In Italy, at the same date, there were 12,462 cases of infection and 827 deaths in total. At the time of the preparation of this report, the number of infected individuals had increased to > 1.2 million worldwide. COVID-19 has quickly become a global threat to public health.

## Case presentation

**Fig. 1:**
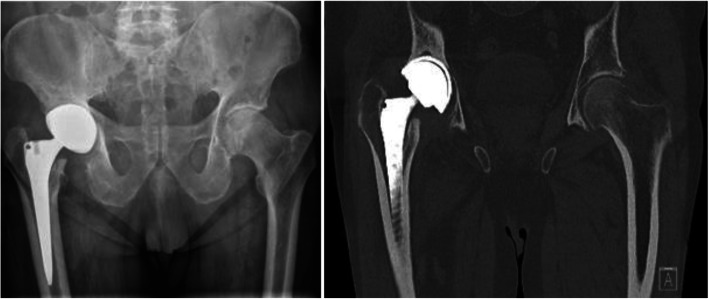
A-P X-Ray and CT-Scans showed the loosening of the prosthesis

The patient presented himself in our orthopedic clinic 4 years after the implantation of a right hip total prosthesis. The patient was 78 years old, with a history of stage IIIA B cell lymphoma in remission. He had been limping with severe pain for about 2 months, walking with crutches, with an about 2-cm difference in the lower limbs. Blood tests showed 11.4 × 1000/μL leukocytes, CRP at 7.53 mg/dL and ESR at 84 mm/1 h. X-rays and CT scans showed signs of loosening of the prosthesis towards the pelvis. (Fig. 1)*.*

Before performing a revision, we decided to make a sterile puncture in the operating room under C-arm fluoroscopic control. While waiting for the result of the antibiogram, the patient was treated with an empirical antibiotic therapy. After finding the presence of *E. coli*, the patient was treated with Cefotaxim 2 g three times per day for 6 weeks.

**Fig. 2:**
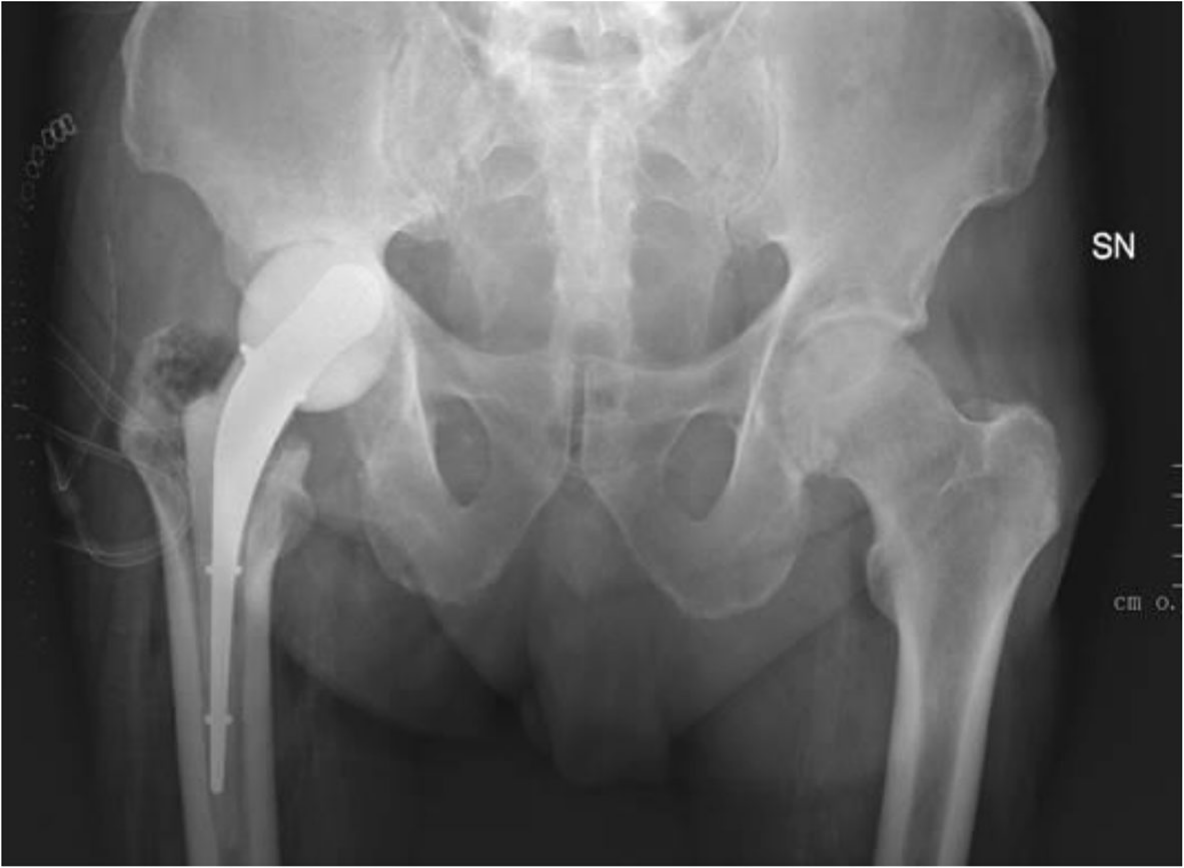
Removal of the prosthesis and implant of the spacer

Then, we decided to remove the implanted prosthesis and to substitute with antibiotic-impregnated cement spacers (Gentamycin and Vancomycin). The femoral stem, the acetabular component and swabs of the muscular fascia and synovial joint fluid were sent to microbiological laboratory for further analysis and the test did not reveal growth of any germ. For this reason, after adequate monitoring of the patient’s general condition, he was discharged with an oral antibiotic therapy prescribed and full weight bearing, since the pain was tolerable. (Fig. 2).

According to guidelines, the patient should have received the implantation of the final prosthesis after 6 weeks. However, during the same period, the COVID-19 pandemic occurred and, therefore, in order to plan the definitive operation, it was necessary to contact the hospital’s Task Force, which allowed it only 9 weeks after the spacer placement [2–4].

**Fig. 3:**
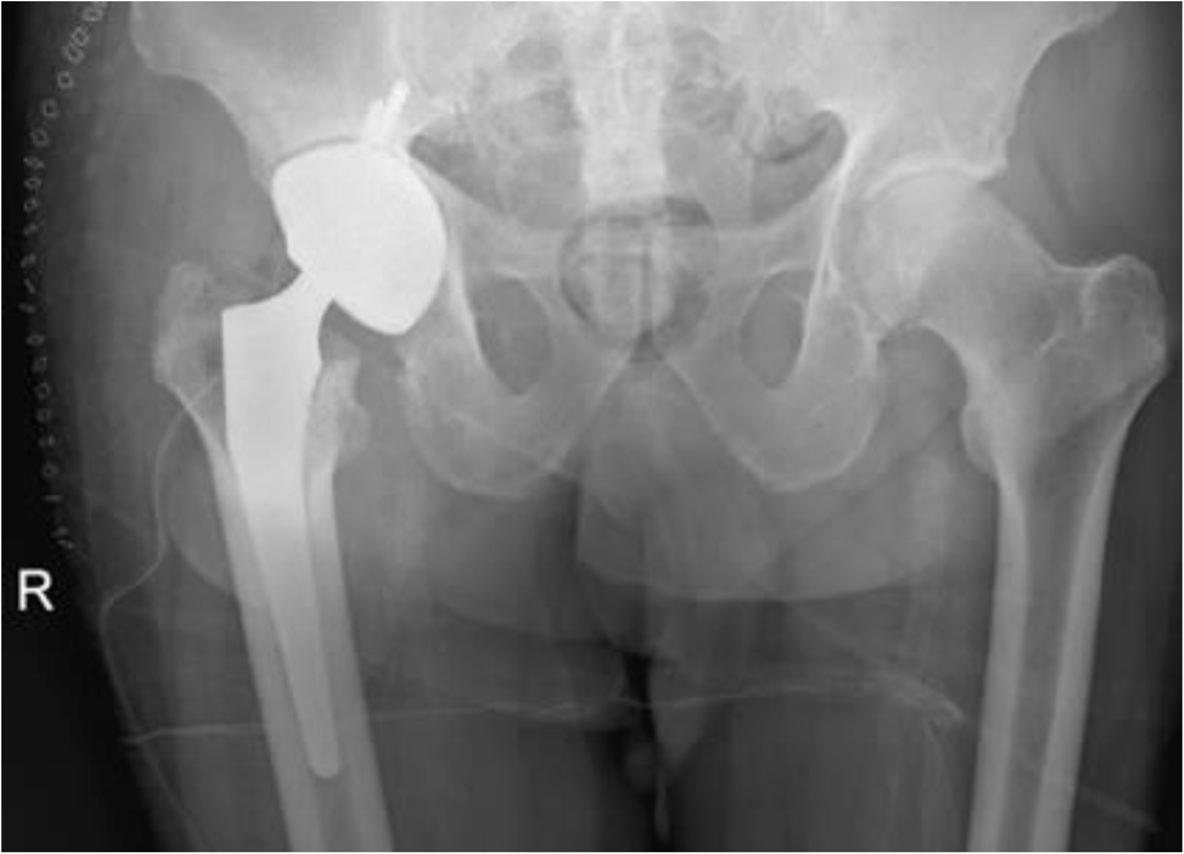
Definitive prosthesis after 9 weeks

After implantation of the final prosthesis (Acetabular Component 62, Delta TT Company Lima Corporate and two screws 6.5 mm, Stem 12 LCU Company Link, small head 36 mm Ceramic), the postoperative course was uneventful, and the patient remained asymptomatic, except for a mild anemia, which was treated with Ferric sodium gluconate for 1 week. The postoperative prophylaxis of the infection included a double antibiotic protocol with Cefotaxim 2 g three times per day and Rifampicin 600 mg once in the evening for the following 8 weeks. (Fig. 3).

**Fig. 4:**
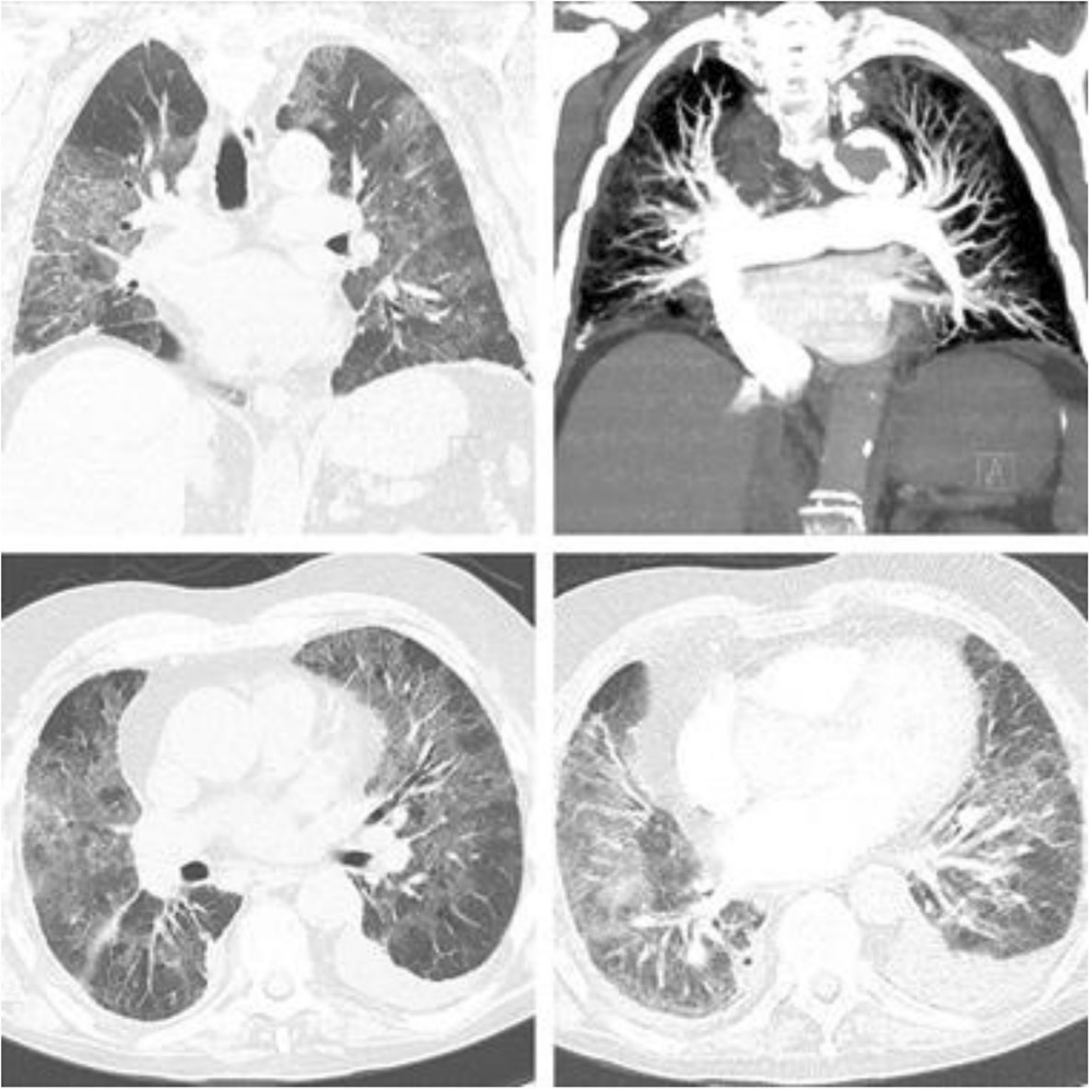
Thorax CT-Scan showed multiple areas of ground-glass opacification

During his second hospital stay, two nasal-pharyngeal swab tests were performed for COVID-19, both being negative. The patient was asymptomatic for the first postoperative week. However, while the patient was in our department on the eighth postoperative day, after a sudden worsening of the respiratory symptoms with low oxygen saturation (SpO_2_ 87%) and severe respiratory distress, an emergency chest X-ray and then a CT scan were performed, and they showed no evidence of pulmonary embolism but revealed multiple areas of ground-glass opacification (GGO) (Fig. 4).

The wound looked normal without signs of infections or dehiscence and there was no swelling or hematoma on the operated hip. We decided to perform a COVID-19 rapid test, which showed that patient was positive for COVID-19 IgM.

According to the literature [5, 6], there are no defined radiological criteria to distinguish between a pulmonary embolism and a Sars Cov-2 Infection. Moreover, pulmonary embolism (PE) is seen in a high frequency in hospital-treated patients with COVID-19, with an incidence of 30%.

At this time, we had to decide where we had to transfer our patients. As part of a Health Care Company made by 7 Hospitals, the designated Task Force decided to divide the Patients into two subtypes:

1. Patient who tested positive for COVID-19 (Swab test) and had clinical symptoms and/or radiological evidence of COVID-19 Disease;
2. Patient whose results of COVID-19 Swabs were negative but had clinical symptoms or radiological evidence for COVID-19 Disease.

The two different groups were treated similarly but in different isolation departments.

Therefore, the patient was transferred to the second one, waiting for further swab tests and further treatment. One week later, after the resolution of the pulmonary symptoms, the patient was finally discharged home.

Two subsequent check-ups were carried out 6 and 10 weeks after surgery, with the first one including X-ray examination. In both examinations, the patient did not report pain, the wound was dry and clean, the mobility was good and the radiography demonstrated an excellent position of the prosthesis without signs of detachment. Blood tests were consistently normal, with stable CRP level.

## Discussion

In order to reach the final diagnosis, all the steps required by the literature [7] were taken, including: the pre-operative anamnestic and laboratory evaluation, the arthrocentesis, the two-step revision and antibiotic therapy for 6 weeks, adapted on the basis of antibiogram [8].

Treatment depends on the stage of infection and is based on the classification published by Coventry in 1975 with the modification by Tsukayama [9]: Stage I infection occurs acutely within 6 weeks after implantation; Stage II infection refers to delayed chronic presentations; Stage III infection takes place in a previously well-functioning joint replacement; Stage IV infection is unexpected positive culture results in what was thought to be an aseptic revision.

According to the guidelines, the therapeutic scheme in periprosthetic infections for the removal of the implant and the subsequent definitive prosthesis placement is of one or two stages. The last one is considered to be the gold standard with an eradication rate greater than 90% [1, 10].

## Conclusion

The purpose of this case report is to point out the correct treatment of PJI, especially during this pandemic, as evidenced in a patient who developed typical clinical symptoms and showed radiological features of a Sars-Cov-2 infection.

Despite the ongoing COVID-19 pandemic, which delayed the implantation of the definitive prosthesis for a further 3 weeks and the patient’s acute pulmonary distress [11], the periprosthetic infection was eradicated, which was in line with the guidelines in terms of both medical and surgical diagnosis and therapy. As a result, the patient was able to resume his normal daily activity.

## Data Availability

The datasets analyzed during the current study are available from the corresponding author on reasonable request.

## Abbreviations

CT: Computer Tomography
GGO: Ground-glass opacification
CRP: C-reactive protein
ESR: Erythrocyte Sedimentation Rate
PJI: Periprosthetic Joint Infection

## References

[1] Halim A, Grauer JN. Orthopedics in the era of COVID-19; 2020. 10.3928/01477447-20200426-01 32421197

[2] Osmon DR, Berbari EF, Berendt AR, Lew D, Zimmerli W, Steckelberg JM, et al. Diagnosis and Management of Prosthetic Joint Infection: Clinical Practice Guidelines by the Infectious Diseases Society of Americaa. Diagnosis and Management of Prosthetic Joint Infection. 2013. 10.1093/cid/cis803 23223583

[3] Chieffo G, Corsiac S, Rougereauc G, Enserd M, Eyrolled LJ, Kernéisa S, et al. Six-week antibiotic therapy after one-stage replacement arthroplasty for hip and knee periprosthetic joint infection. Med Mal Infect. 2020;50:567. 32284220 10.1016/j.medmal.2020.03.003

[4] Shahpari O, Mousavian A, Elahpour N, Malahias MA, Ebrahimzadeh MH, Moradi A. The Use of Antibiotic Impregnated Cement Spacers in the Treatment of Infected Total Joint Replacement: Challenges and Achievements. Arch Bone Jt Surg. 2020;8(1):11-20. PMID: 32090140; PMCID: PMC7007713. 10.22038/abjs.2019.42018.2141. 10.22038/abjs.2019.42018.2141 PMC7007713 32090140

[5] Bayraktaroğlu S, Çinkooğlu A, Ceylan N, Savaş R. The novel coronavirus pneumonia (COVID-19): a pictorial review of chest CT features. Diagn Interv Radiol. 2020. 10.5152/dir.2020.20304. 10.5152/dir.2020.20304 PMC7963373 32815523

[6] Beckman M, Nyrén S, Kistner A. A case-report of widespread pulmonary embolism in a middle-aged male seven weeks after asymptomatic suspected COVID 19 infection. Thromb J. 2020;18:19. 32868974 10.1186/s12959-020-00235-w PMC7453687

[7] Yung CS, Fok KCH, Leung CN, Wong YW. What every orthopaedic surgeon should know about COVID-19: A review of the current literature. J Orthop Surg (Hong Kong). 2020;28(2):2309499020923499. PMID: 32406305. 10.1177/2309499020923499. 10.1177/2309499020923499 32406305

[8] Senthi S, Munro JT, Pitto RP. Infection in total hip replacement: meta-analysis. Int Orthop. 2011;35(2):253-60. Epub 2010 Nov 18. PMID: 21085957; PMCID: PMC3032119. 10.1007/s00264-010-1144-z. Epub 2010 Nov 18. 10.1007/s00264-010-1144-z PMC3032119 21085957

[9] Tsukayama DTERGR. Infection after total hip arthroplasty. A study of one hundred and six infections. J Bone Joint Surg Am. 1996;78:512–23. 8609130 10.2106/00004623-199604000-00005

[10] Garvin KL, Hanssen AD. Infection after total hip arthroplasty. Past, present, and future. J Boint Surgery Am. 1995;77:1576. 10.2106/00004623-199510000-00015 7593069

[11] De Caro F, MH T, Verdonk P. Returning to orthopaedic business as usual after COVID-19: strategies and options. Knee Surg Sports Traumatol Arthrosc. 2020;28:1699. 32342140 10.1007/s00167-020-06031-3 PMC7185264

